# Cerebral capillary oxygen diffusion: exploring the concept of intracapillary hemoglobin conformational changes

**DOI:** 10.1186/s40635-024-00691-y

**Published:** 2024-11-28

**Authors:** Gurgen Harutyunyan, Varsenik Harutyunyan Jaghatspanyan, Garnik Harutyunyan Jaghatspanyan, Emma Martirosyan, Artur Cherkezyan, Armen Varosyan, Suren Soghomonyan

**Affiliations:** 1 Hospital 9 de Octubre, VITHAS, Valle de La Ballestera 59, 46015 Valencia, Spain; 2 Faculty of Pharmacy, Universitat de València, C. del Cementerio, 1Burjassot, 46100 Valencia, Spain; 3 Faculty of General Medicine, Yerevan State Medical University, 2 Koryun St, 0025 Yerevan, Armenia; 4 Erebouni Medical Center, Titogradyan St. 14, 0087 Yerevan, Armenia; 5 The Ohio State University, Wexner Medical Center N411 Doan Hall, 410 West 10Th Avenue, Columbus, OH 43210 USA

**Keywords:** Brain tissue oxygen pressure, Oxygen diffusion, Relaxed and tense state hemoglobin, Hyperoxia, Hill coefficient

## Abstract

The mechanisms of oxygen diffusion in brain capillaries have not been fully clarified to date. According to the laws of physics, the well-documented phenomenon of hyperoxemia-induced excessive increases in brain tissue oxygen pressure (PbtO2) contradicts traditional models of cerebral capillary oxygen diffusion. Circulating models predict a significant drop in oxygen pressure (PO2), and some of them foresee the presence of hypoxic or anoxic corners near the capillary end, regardless of high PbtO2 levels. We propose that the cerebral intracapillary transformation of hemoglobin from the relaxed (R) to the tense (T) quaternary conformational state, driven by deoxygenation and an overload of negative allosteric effectors, and characterized by a lower, more hyperbolic dissociation curve, mitigates the oxygen pressure difference across cerebral capillaries, ensuring a homogeneous pericapillary distribution of oxygen. The hemoglobin R to T state transition is responsible for the high PbtO2 levels observed in viable cerebral tissue during hyperoxemia.

## Introduction

Transportation of oxygen to brain tissue involves complex physiological processes to ensure a continuous and adequate oxygen supply. Various models have been developed to simulate and predict oxygen diffusion in the brain capillaries. Krogh devised a simplistic geometric model that illustrates the basic skeletal muscle tissue unit (a cylinder) supplied by a single capillary. According to this model, the intracapillary oxygen PO2 drops significantly as blood traverses the capillary, allowing for hypoxic or anoxic corners located in the far area of the capillary end [1]. To avoid an unrealistic mosaic distribution of oxygen, several authors have adapted models to more accurately reflect the uniformity of oxygen concentration across brain tissue. Lücker and colleagues [2] proposed a higher capillary density on the venular side to ensure tissue oxygenation. Metzger [3] proposed a cubic-lattice model for a brain microcirculatory unit. Kislyakov and colleagues [4] and Grunewald and Sowa [5] suggested four-capillary microcirculatory unit. These and many other similar models assume a consistently high Hill coefficient (n) for hemoglobin in brain capillaries (typically between 2.7 and 3). The high Hill coefficient indicates a predominant relaxed (R) quaternary conformational state of hemoglobin along brain capillaries and underscores the sigmoidal nature of the oxyhemoglobin dissociation curve (ODC), leading to a significant decrease in end-capillary PO2 by hemoglobin desaturation. Furthermore, hemoglobin in the R state lacks the sufficient buffering capacity to neutralize the acidity generated by brain metabolism.

The ability of hemoglobin to transition from the R to the T state in response to deoxygenation and increased concentrations of negative allosteric effectors (e.g., 2,3-diphosphoglycerate, CO2, H + , Cl −) is a biological adaptation to varying metabolic conditions. Our concept suggests that the intracapillary transition of hemoglobin from the R to the T state, coupled with a gradual decrease in the Hill coefficient from 2.7 to 1 or lower, results in a lower, more hyperbolic ODC and a significant reduction in hemoglobin's affinity for oxygen. This shift ensures a nearly uniform distribution of oxygen both within and around the capillaries.

### Concept base and description

The proposed concept for oxygen diffusion in cerebral capillaries is firmly rooted in biochemistry, physiology, and physics, and is further validated by clinical data. Hemoglobin with high oxygen affinity starts releasing oxygen upon entering the capillary. As it moves along the capillary, the concentrations of deoxyhemoglobin and negative allosteric effectors increase, causing a decrease in both the Hill coefficient and hemoglobin-oxygen affinity. Consequently, the sigmoidal ODC gradually shifts rightward along the capillary, transforming into a lower hyperbola as the Hill coefficient drops to one or lower [6]. This transition aligns with the shift from the R to the T state of hemoglobin's quaternary structure, an essential adaptive process that maintains a nearly uniform PO2 both within and around the capillaries, even as hemoglobin oxygen saturation (SO2) significantly decreases. Furthermore, only the enhanced buffering capacity of the T state enables the neutralization of acidity resulting from CO2 buildup in high-metabolism brain tissue with a high respiratory quotient (RQ) of one [6].

**Fig. 1:**
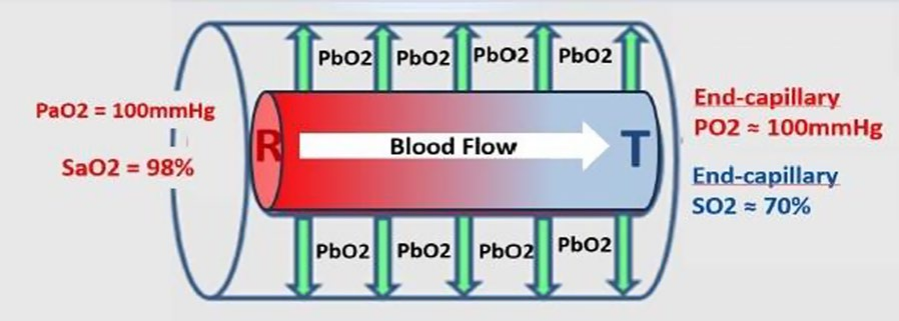
Transition of hemoglobin from relaxed (R) to tense (T) state in cerebral capillaries: Assumed homogeneous pericapillary distribution of oxygen pressure (PbO2). PO2—intracapillary oxygen tension, SO2—oxygen saturation of hemoglobin

**Fig. 2:**
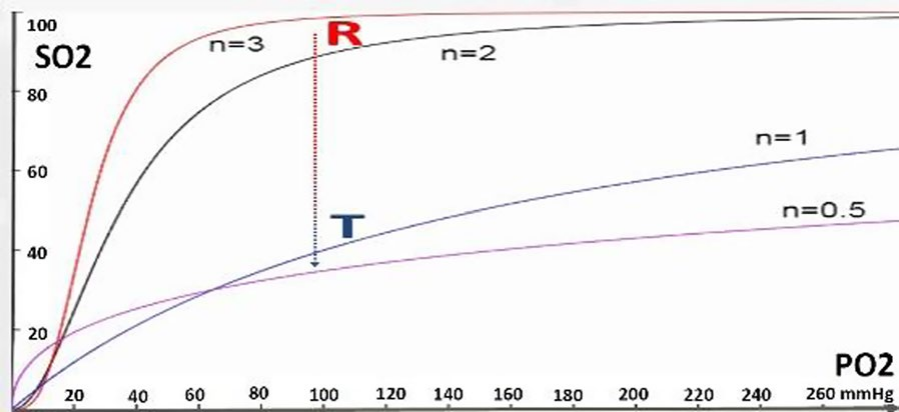
Didactic dissociation curves illustrate both the relaxed (R) and tense (T) states of hemoglobin. The transition from the R to the T state through desaturation (arrow) does not result in a significant change in intracapillary oxygen tension (PO2). n—Hill coefficie

In other words, the presence of hemoglobin in the T state at the cerebral capillary end is crucial for achieving homogeneous oxygen diffusion and effective elimination of metabolic waste products—processes that are not possible when hemoglobin remains in the R state due to its biochemical properties. The well-known hyperoxemia-induced excessive increase in PbtO2 exemplifies the presence of hemoglobin in the T state at the cerebral capillary end. This state is characterized by a lower hyperbolic ODC, where a significant reduction in SO2 due to a high oxygen extraction fraction (OEF) is accompanied by a high PO2. (Figs. 1 and 2).

### Clinical evidence

The transition of hemoglobin from the R to the T state within brain capillaries is supported by clinical data obtained through neuromonitoring. The excessive increase in PbtO2 due to normobaric hyperoxia is widely recognized through the routine tests conducted to verify the proper functioning of the PbtO2 electrode. McLeod et colleagues [7] demonstrated a substantial elevation in PbtO2, measuring 147 ± 36 mmHg during hyperoxemia. Similarly, Gargadennec and colleagues [8], in patients with traumatic brain injury (TBI) without hypoperfusion, reported PbtO2 levels of 123 (96–138) mmHg, consistent with previously published results by Rosenthal and colleagues [9], who observed levels of 122 mmHg. Vidal-Jorge and colleagues [10] noted relatively moderate PbtO2 levels under normobaric hyperoxia, with increases up to 54 and 56 mmHg in relatively healthy areas and in zones of traumatic penumbra, respectively. In some cases, PbtO2 can reach up to 250 mmHg when the inspired fraction of oxygen is set to one (unpublished data).

Cerebral near-infrared spectroscopy (NIRS) readings during hyperoxemia typically indicate a minimal increase in regional oxygen saturation (rSO2) of 2.8 ± 1.82% [7], or show no changes in rSO2 in tissues with intact autoregulation [11]. Moreover, clinical studies using positron emission tomography and magnetic resonance imaging (MRI) have consistently demonstrated a high OEF in non-necrotic tissue even under hyperoxia [7, 11–14]. For instance, Engle and colleagues [15] reported a mean OEF value of 38.71% in a meta-analysis of 26 studies involving 349 patient data points. These findings suggest that during hyperoxemia, hemoglobin at the capillary end, despite exhibiting low SO2 due to a high OEF, is exposed to an environment with very high PO2. While the methods mentioned above provide either global or local measurements and refer to different regions of assessment, we can infer that in the area where the PbtO₂ electrode is placed, hemoglobin at the capillary end becomes significantly desaturated due to the high OEF, despite the very high PO₂ in the surrounding environment during hyperoxemia. In other words, the prevalence of the T state of hemoglobin at the cerebral capillary-end, characterized by a reduced and more hyperbolic ODC, is undeniable. In other words, we can assume that the predominance of the T state of hemoglobin at the cerebral capillary end, characterized by a reduced and more hyperbolic ODC, is highly likely.

## Discussion

The two-state model of hemoglobin, developed by Perutz and colleagues in the 1960s, [16] has yet to be fully applied in the context of neurotrauma. However, applying the concept of hemoglobin's conformational changes from the R to the T state within cerebral capillaries provides a clear explanation for the homogeneous intra- and pericapillary distribution of oxygen in brain tissue, the exaggerated increase in PbtO2 induced by hyperoxemia, and other important processes that we have discussed in detail elsewhere [17, 18]. In the R state, as is currently accepted, hemoglobin with low SO2 at the capillary end would be expected to exhibit decreased PO2. Consequently, oxygen diffusion in the capillaries would align with the Krogh model, resulting in the presence of hypoxic or anoxic regions. Moreover, the very low buffering capacity of the R state would significantly limit cerebral metabolism. This single-state model of R hemoglobin, used to explain oxygen diffusion in brain capillaries, raises both practical and theoretical challenges in the context of neurotrauma.

In recent years, an intriguing discussion has emerged regarding the explanation for the genesis of high PbtO2 during normobaric hyperoxia. To address this phenomenon, Gargadennec and colleagues [8] proposed an "increase in interstitial oxygen diffusion at the arterial capillary side." Meanwhile, Hoiland and colleagues [19] put forward a bold hypothesis suggesting that the PbtO2 catheter might be unable to distinguish between the dissolved oxygen tension in the brain parenchyma and the microvasculature. According to Ercole [20], increasing PaO2 may enhance PbtO2 as a spatial average across tissue but does little to improve oxygen tension in the hypoxic fraction of tissue.

As can be seen, all three explanations imply an unchanged R state of hemoglobin in the capillaries. Despite the high desaturation and accumulation of negative allosteric effectors, these interpretations overlook the prevalence of the T state of hemoglobin at the capillary-end of cerebral capillaries. The volume of the Licox (with a diameter of 0.8 mm and a length of 5 mm) or Neurotrend (with a diameter of 1.65 mm and a length of 5.5 mm) electrodes' PbtO2 measurement chambers exceeds that of a cerebral capillary (diameter of 6 µm, length 0.5 mm) by more than 100,000 times. Thus, the equivalent volume of nearby brain tissue surrounding the oxygen chamber would have a PO2 equal to or greater than the measured PbtO2; Otherwise, oxygen reflux would occur. According to the laws of physics, PbtO2 readings represent the lowest recorded PO2 within a relatively large brain tissue area encompassing thousands of capillaries around the PbtO2 electrode. Consequently, PbtO2 cannot be simply derived from the arterial capillary side PO2 and cannot be considered a spatial average value, as there is no oxygen source within the relatively large volume of the oxygen measurement chamber. This value can only be balanced with the capillary-end PO2 and represents the minimum PO2 within the area where the PbtO2 electrode is placed. Additionally, manufacturers' data indicate an accuracy of ± 2.5 mmHg for PbtO2 levels below 120 mmHg, suggesting that PbtO2 electrodes provide reliable measurements within this range.

According to the concept of hemoglobin's intracapillary transition from the R to T state, PbtO2 is a derivative of the venous-end capillary PO2, which aligns with the previously accepted hypothesis by Johnston and colleagues [21], although some co-authors of that study have since changed their position against it.^8^ A deeper understanding of cerebral capillary oxygen diffusion and the genesis of PbtO2 will significantly enhance the value and importance of this monitoring in neurotrauma, while also helping to prevent related miscalculations and errors.

## Conclusion

The cerebral intracapillary transformation of hemoglobin from the relaxed to the tense quaternary conformational state is crucial for maintaining cerebral metabolism. The tense state, with its distinct biochemical properties, helps reduce the oxygen pressure gradient across cerebral capillaries, thereby ensuring a homogeneous distribution of oxygen in the surrounding brain tissue.

## Data Availability

All data generated or analyzed during this study are included in this published article. Any additional datasets used and/or analyzed during the current study are available from the corresponding author upon reasonable request.

## Abbreviations

PbtO2: Brain tissue oxygen pressure
PaO2: Arterial oxygen pressure
R: Relaxed hemoglobin
T: Tense hemoglobi
PO2: Oxygen pressure
n: Hill coefficient
ODC: Oxyhemoglobin dissociation curve
SO2: Oxygen saturation of hemoglobin
RQ: Respiratory quotient
NIRS: Near‐infrared spectroscopy
Rso2: Regional oxygen saturation
mri: Magnetic resonance imaging
OEF: High oxygen extraction fraction
